# Focal Neurological Symptoms at Initial Presentation Could Be a Potential Risk Factor for Poor Prognosis Among Patients With Multiple Brain Abscesses by Streptococcus anginosus Group: A Case Report With Literature Review

**DOI:** 10.7759/cureus.32085

**Published:** 2022-11-30

**Authors:** Natsuki Shibamura, Daisuke Miyamori, Terumasa Tanabe, Naoto Yamada, Susumu Tazuma

**Affiliations:** 1 General Internal Medicine, JA Onomichi General Hospital, Onomichi, JPN; 2 General Internal Medicine, Hiroshima University Hospital, Hiroshima, JPN; 3 Emergency, JA Onomichi General Hospital, Onomichi, JPN; 4 Neurosurgery, JA Onomichi General Hospital, Onomichi, JPN; 5 Internal Medicine, JA Onomichi General Hospital, Onomichi, JPN

**Keywords:** streptococcus anginosus, focal neurological deficits, prognostic indicators, neurologic prognosis, literature review, multiple brain abscesses

## Abstract

*Streptococcus anginosus* group (SAG) is one of the most common microbes of brain abscesses. Brain abscesses caused by SAG have often delayed diagnosis since both blood and cerebrospinal fluid cultures are negative in half of the cases.

A 68-year-old man developed persistent fever, headache, and myalgias for two weeks and visited our department. He was treated with oral antibiotics without laboratory work. Although examination showed no focal neurological symptoms, a careful interview revealed a history of unusual behavior for a few minutes on the previous day. Whole body contrast-enhanced computed tomography (CT) and head magnetic resonance imaging (MRI) showed two ring enhancements close to the bilateral ventricles, which were consistent with a diagnosis of the brain abscesses. An emergent surgical puncture for the larger abscess with intravenous antimicrobial therapy quickly improved his condition, and he was discharged on day 36 with no sequelae.

We retrospectively reviewed works of literature on cases with multiple brain abscesses by SAG to assess potential prognostic factors for neurological sequelae. Statistical analyses of 12 cases, including 11 cases from the literature review and the current case, were performed between groups with or without poor prognosis. Among potential risk factors of age, sex, focal neurological symptoms, duration from onset to treatment, abscess formation of other organs, presence of surgical drainage, and positive for blood culture, only focal neurological symptoms at the initial presentation were significantly associated with poor prognosis (no poor prognosis, 1/4 cases vs poor prognosis group, 8/8 cases; p=0.01).

Careful interviews and detailed examinations should be conducted to assess the possibility of brain abscesses among patients with fever of unknown etiology. Otherwise, a delayed diagnosis might result in poor prognoses such as death or neurological sequelae due to this disease's nature, which has few specific symptoms in the early stages.

## Introduction

Reports of abscess formation caused by *Streptococcus anginosus* group (SAG) have recently increased possibly due to the antibiotic microbial resistance measures and vaccine-induced changes in colony formation [1-4]. SAG colonize in the oropharynx, gastrointestinal tract, and genitourinary tract, and causes brain abscesses by direct spread from head and neck infection or hematogenous spread from systemic organs [5,6]. Directly transmitted lesions tend to form a single abscess, while hematogenous transmitted lesions tend to develop multiple abscesses. Since blood and cerebrospinal fluid cultures are negative in one-third and half of the cases, respectively [4], there have been reports of cases with sequelae due to delayed diagnosis [7]. We report a case of multiple brain abscesses caused by SAG, which were successfully treated without any neurological sequelae due to the early diagnosis and emergent surgical drainage. Our literature review identified 11 similar cases. We assess the potential risk factor for neurological sequelae among patients with multiple brain abscesses by SAG.

## Case presentation

A 68-year-old man presented to our hospital with a persistent fever. He had a history of hypertension, dyslipidemia, and type 2 diabetes mellitus, but denied any primary headaches nor a history of dental treatment. For two weeks, he had been suffering from sub-febrile of high 37 degrees Celsius almost every day. Headache and myalgia in proximal muscles had appeared concurrently. Twelve days before our department visit, he visited a general practitioner for the same symptom and received a ceftriaxone (CTRX) 1g infusion and was prescribed levofloxacin (LVFX) 500 mg/day for nine days. His vital signs, including a body temperature of 37 degrees Celsius, were stable. He was conscious and did not complain of any specific symptoms at the time of examination. However, after conducting a detailed medical history interview, the wife informed us that the patient had exhibited unusual behavior the previous day in which he searched for his mother, who had died in the past. He complained of fatigue in the extremities, but physical examination revealed no obvious focal neurological symptoms including meningeal irritation signs or peripheral signs. Laboratory tests (Table 1) showed a normal white blood cell count and mildly elevated C-reactive protein.

**Table 1:** Laboratory workup. H, high; L, Low; hr, hour; WBC, white blood cell.

Lab	Value	Reference Range
Complete Blood Count		
WBC Count	6320	3300-8800/μL
%Neutrophils	68.2	35-75%
%Lymphocyte	20.8 L	25-55%
Red Blood Cell	500	435-555x10^2^/μL
Hemoglobin	16.4	13.7-16.8g/dL
Hematocrit	48.2	40.7-50.1%
Mean Corpuscular Volume	96.5	83.6-98.2fl
Platelet Count	31.0x10^4^	15.8-34.8/μL
Biochemical Test		
Total Protein	7.7	6.6-8.1g/dL
Albumin	4.3	4.1-5.1g/dL
Total bilirubin	0.56	0.40-1.50mg/dL
Aspartate Aminotransferase	26	13-30U/L
Alanine Aminotransferase	27	10-42U/L
Alkaline Phosphatase	65	38-113U/L
γ-Glutamyl Transpeptidase	31	13-64U/L
Lactate Dehydrogenase	135	124-222U/L
Blood Urea Nitrogen	18	8.0-20.0mg/dL
Creatinine	0.83	0.65-1.07mg/dL
Sodium	137 L	138-145mEq/L
Potassium	4.8	3.6-4.8mEq/L
C-Reactive Protein	1.54 H	below 0.14mg/dL
Procalcitonin	0.14	below 0.5ng/mL
Blood Glucose	94	70-100 mg/dL
HbA1c	7.3	below 6.5%
Blood Sedimentation Rate	28 H	3-10mm/hr
Cerebrospinal Fluid Test		
Open Pressure	230 H	60-150 mmH2O
Cell Count	592 H	0-5/μL
Mononucleosis	385 H	0-5/μL
Polynuclear	207 H	0-5/μL
Protein	93 H	10-40mg/dL
Chlorine	117 L	120-128mEq/L
Glucose	58	50-80mg/dL

**Figure 1:**
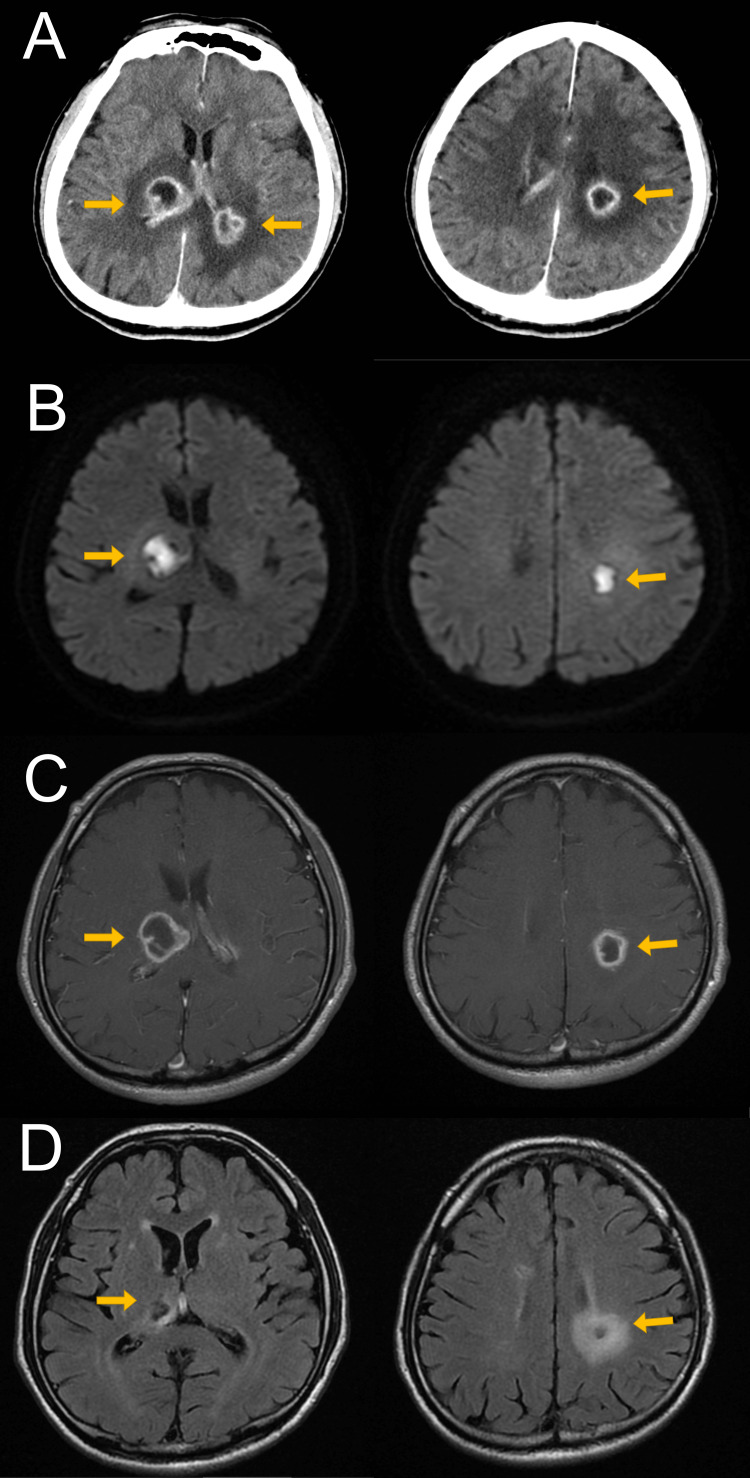
Brain computed tomography and magnetic resonance imaging (A) Contrast-enhanced CT of the brain at the time of admission shows the ring-enhanced low-density area (arrows). (B) Diffusion-weighted MRI of the brain at the time of admission shows a marked high-signal area (arrows). (C) Contrast-enhanced MRI images of the brain at the time of admission show a uniform high-signal capsule and an internal low-signal area (arrows). (D) Contrast-enhanced MRI images of the brain at the time of discharge show a size reduction in both brain abscesses (arrows).

Contrast-enhanced CT (Figure 1A) showed two ring-enhanced nodules (diameter; right: 23.0 mm, left: 17.6 mm) with internal uniform low-density area close to the bilateral ventricles. The surrounding area also showed a low-density area. There were no findings in the thorax or abdomen that could be a cause of infection. Transthoracic echocardiography showed no obvious vegetation. Cerebrospinal fluid examination (Table 1) showed an increased cell count with mononuclear cell predominance, without any evidence of bacterial organisms on Gram staining. MRI showed a marked high-signal area on the diffusion-weighted imaging (Figure 1B), consistent with the ring-enhanced nodules on the CT scan. Contrast-enhanced MRI (Figure 1C) showed a uniform high-signal capsule and an internal low-signal area.

**Figure 2:**
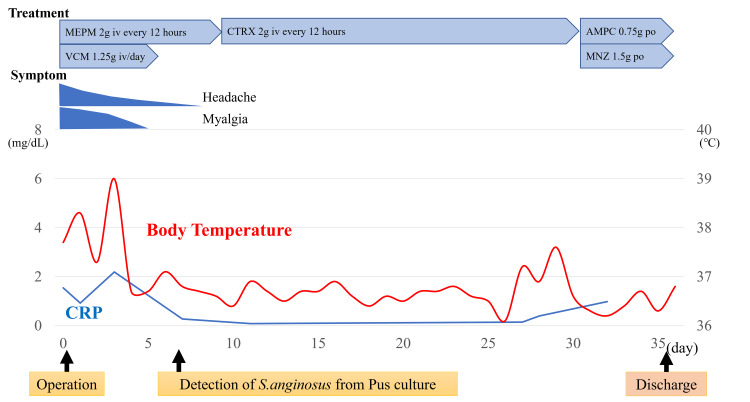
Clinical course of the patient After the diagnosis of multiple brain abscesses, meropenem 2g every 12 hours and vancomycin 1.25g per day was initiated. De-escalation was conducted with ceftriaxone 2g every 12 hours after the result of the microbial sensitivity test. He was discharged on the 36th day of admission with the prescription of amoxicillin 250mg and metronidazole 500mg orally every 8 hours. Headache, myalgia, body temperature, and CRP improved in several days. MEPM: meropenem, VCM: vancomycin, CTRX: ceftriaxone, AMPC: amoxicillin, MNZ: metronidazole, S. anginosus: Streptococcus anginosus, CRP: C-reactive proteins.

Figure 2 shows the clinical course of the patient. Based on the clinical and imaging findings, we diagnosed him with multiple brain abscesses. We consulted a neurosurgeon on the same day. Subsequently, they performed urgent surgical drainage and punctured the right brain abscess, which was the larger one, and aspirated pus of 5 ml. Meropenem 2 g every 12 hours and vancomycin 1.25 g per day were administered from the day of admission. SAG was detected in the culture of aspirated pus on the sixth postoperative day, and a follow-up MRI showed the shrinkage of the brain abscesses on both sides, thus the antibiotics were de-escalated to CTRX 2g every 12 hours according to the result of the microbial susceptibility test (Table 2). Blood and spinal fluid cultures were negative. Laboratory findings improved promptly after treatment.

**Table 2:** Result of microbial susceptibility test MIC: Minimal inhibitory concentration.

Antibiotics	MIC	Susceptibility
Benzylpenicillin (PCG)	<=0.03	susceptible
Ampicillin (ABPC)	<=0.06	susceptible
Cefotiam (CTM)	2	not available
Cefotaxime (CTX)	<=0.12	susceptible
Ceftriaxone (CTRX)	0.25	susceptible
Cefozopran (CZOP)	0.5	susceptible
Cefepime (CFPM)	<=0.5	susceptible
Cefditoren (CDTR)	<=0.06	not available
Meropenem (MEPM)	<=0.12	susceptible
Amoxicillin / Clavulanate (CVA/AMPC)	<=0.25	not available
Erythromycin (EM)	<=0.12	susceptible
Azithromycin (AZM)	<=0.25	susceptible
Minocycline (MINO)	<=0.5	susceptible
Levofloxacin (LVFX)	0.5	susceptible
Vancomycin (VCM)	1	susceptible
Sulfamethoxazole/Trimethoprim (ST)	<=0.5	not available
Chloramphenicol (CP)	<=4	susceptible
Rifampicin (RFP)	<=1	not available

During hospitalization, the patient progressed with no focal neurological symptoms. Since MRI (Figure 1D) showed improvement in both brain abscesses, the patient was discharged on the 36th postoperative day. Three months after discharge, there was no recurrence of the brain abscess, scarring of the right abscess, and further shrinkage of the left abscess.

## Discussion

We experienced a case of multiple brain abscesses caused by SAG that was discharged from the hospital without neurological sequelae due to early diagnosis and drainage. In this case, there was no evidence of infection in the head and neck region, and there was no history of dental treatment, thus the route of bacterial invasion was unknown. The patient had been treated with antimicrobial agents at another hospital before admission without performing culture tests, and partial treatment may have improved the bacteremia, but only exacerbated the brain abscess due to inadequate duration of treatment.

SAG was the cause of brain abscess in 30-74% of the cases in which a pathogen could be identified [4,8,9]. Mortality from brain abscesses has been reported to be significantly associated with age, multiple abscesses, immunosuppression, and coexisting cardiac disease [9]. However, the neurological prognosis of multiple brain abscesses has not been studied. The etiology of single brain abscess and multiple ones are potentially different and clinical symptoms of multiple brain abscesses by SAG developed more gradually than other pathogens, such as *Staphylococcus aureus*. In this study, we reviewed the present case and previous literature regarding poor prognostic factors related to neurological sequelae among multiple brain abscesses caused by SAG using PubMed with the search term “multiple brain abscesses”, “*Streptococcus milleri*”, “*Streptococcus intermedius*” and “*Streptococcus anginosus*”. We include all the cases with multiple brain abscesses caused by SAG from the search result [5,10-15]. Cases with epidural, subdural abscesses, or unknown neurological sequelae were excluded from the review. Twelve cases, including 11 cases from seven previous reports and current case, were included, and eight had focal neurological symptoms such as impaired consciousness or paralysis from the initial diagnosis (Table 3). Blood cultures were negative in two-fifth of the cases. Two patients were presenting after their generalized seizure had resolved, and no focal neurological deficits were noted on physical examination.

**Table 3:** Reviews of cases with multiple brain abscesses. M: male; F: female; +: positive; -: negative.

Author	Year	Age	Sex	Time to diagnosis	Abnormal neurological findings at initial diagnosis	Blood culture	Non-brain abscess	Surgical drainage	Sequelae
Melo and Raff [15]	1978	69	M	4 weeks	Aphasia	+	liver (single)	+	Deceased
Melo and Raff [15]	1978	27	M	3 weeks	Hemianopsia	-	Negative	+	Visual deficit
Melo and Raff [15]	1978	18	M	4 months	Unconsciousness	-	liver (single)	+	Disoriented
Mofredj [14]	1999	60	M	2 days	Right paraplegia/unconsciousness	+	liver (multiple)	-	Right-hand paralysis
Lee et al. [13]	2005	58	M	1 month	Gait disorder/unconsciousness	+	liver (single)	+	Deceased
Wagner et al. [12]	2006	39	M	Unknown	Facial paralysis	+	liver (single)	+	7^th^ cranial nerve palsy
Petti et al. [5]	2008	16	F	5 days	Unconsciousness	-	lung (single)	+	Behavioral abnormalities
Petti et al. [5]	2008	6	M	6 days	Papilledema	-	Negative	+	Behavioral abnormalities
Petti et al. [5]	2008	21	M	5 weeks	Slurred speech	-	Negative	+	None
Erne et al. [11]	2010	61	M	2-3 weeks	Negative (seizure)	-	liver (single)	+	None
Maliyil et al. [10]	2011	21	M	1 week	Negative (seizure)	+	spleen (single)	+	None
Shibamura et al. (current case)	2022	68	M	2 weeks	Negative	-	Negative	+	None

Two patients died and six had neurological sequelae such as hemiplegia, aphasia, and coma. We compared the potential risk factors (age, sex, focal neurological symptoms at the initial presentation, duration from onset to treatment, abscess formation of other organs, presence of surgical drainage, and positive test of blood culture) associated with poor prognosis between cases that resulted in death or sequelae and those that did not have any neurological sequelae (Table 4).

**Table 4:** Comparison of case characteristics with and without sequelae * Wilcoxon rank-sum test, ¶ Fisher-exact test, IQR: Interquartile range.

		Deceased or Sequelae		
	Total	Negative	Positive	p-value
	N=12	N=4	N=8	
Age, median (IQR)	33 (20-61)	41 (21-65)	33 (17-59)	0.50*
Sex, N (%)	11 (92%)	4 (100%)	7 (88%)	1.00*
Etiologic agent, N (%)				1.00¶
S. anginosus	3 (25%)	1 (25%)	2 (25%)	
S. constellatus	1 (8%)	0 (0%)	1 (13%)	
S. intermedius	8 (67%)	3 (75%)	5 (63%)	
Time to diagnosis (days), median (IQR)	18 (6-30)	16 (11-27)	20 (5-30)	0.85*
Focal neurological symptoms at initial diagnosis, N (%)	9 (75%)	1 (25%)	8 (100%)	0.018 ¶
Abscess of non-brain organs, N (%)	8 (67%)	2 (50%)	6 (75%)	0.55¶
Blood culture positive, N (%)	5 (42%)	1 (25%)	4 (50%)	0.58¶

We include 12 cases with multiple brain abscesses by SAG, and the sample size was not enough to analyze multivariate analysis, therefore we performed analysis using the Fisher exact or Wilcoxon rank-sum test. Analysis showed that patients with focal neurological symptoms at presentation were significantly associated with poor prognosis. On the other hand, age and abscesses in other organs, as noted in previous studies, did not show significant differences.

Among patients with multiple brain abscesses caused by SAG, the presence of focal neurological symptoms at initial presentation was a potential prognostic factor of poor prognosis and neurological sequelae. In this case, the diagnosis was confirmed and treatment was initiated before the neurological symptoms appear, which might enable the patient to be discharged from the hospital without any neurological sequelae.

## Conclusions

We experienced a case of multiple brain abscesses caused by *S. anginosus* that was discharged from the hospital without neurological sequelae due to early diagnosis and drainage. The literature review showed the presence of focal neurological symptoms at initial diagnosis was associated with poor prognosis in multiple brain abscesses caused by SAG. When a patient presents with persistent fever, a careful interview should be conducted, and a brain abscess should be listed as a differential diagnosis if any neurological abnormalities or unusual behavior are suspected. Otherwise, due to the nature of this disease, which has few specific symptoms in the early stages, patients would be diagnosed delayed and may have a poor prognosis. Further investigation of prognostic factors is needed in a large series of cases.
